# Traumatic spinal cord injury in Jinan, China: A 10-year hospital-based retrospective observational study of 1134 cases

**DOI:** 10.1097/MD.0000000000049039

**Published:** 2026-05-29

**Authors:** Kun Wang, Linyang Song, Xin Xu, Ang Li, Junlin Han, Ruoxian Song

**Affiliations:** a Department of Orthopaedics, Affiliated 960th Hospital of PLA, Shandong Second Medical University, Jinan, Shandong, China; b Department of Orthopaedics, Qilu Hospital of Shandong University, Jinan, Shandong, China; c Shandong First Medical University (Shandong Academy of Medical Sciences), Jinan, Shandong, China.

**Keywords:** China, electric bicycles, epidemiology, falls, traffic accidents, traumatic spinal cord injury

## Abstract

Traumatic spinal cord injury (TSCI) imposes a substantial clinical and public health burden, yet contemporary epidemiological data from northern China are limited. This study aimed to describe the epidemiological characteristics of TSCI in Jinan, an urban-rural integrated city in northern China. We conducted a hospital-based retrospective observational study of patients with TSCI admitted to the 960th Hospital of the PLA and Qilu Hospital of Shandong University between 2015 and 2024. Medical records of 1134 patients were reviewed. Collected variables included sex, age, marital status, occupation, time of injury, etiology, neurological level of injury, American Spinal Injury Association grade, complications, concomitant injuries, treatments received, and length of hospital stay. Descriptive statistics were used to summarize the data. The mean (standard deviation) age was 62.0 (18.6) years (95% confidence interval, 60.9–63.1), and the male-to-female ratio was 3.69:1. Falls were the leading cause of injury (46.0%), including low-level falls (25.8%) and high-level falls (20.2%), followed by traffic accidents (40.7%). The cervical spine was the most frequently injured region. During hospitalization, 500 of 1134 patients (44.1%) developed complications, most commonly pulmonary infections (16.5%) and urinary tract infections (13.6%). Surgical intervention was performed in 88.9% (1009/1134) of patients. In this 10-year hospital-based series from Jinan, most patients with TSCI were older men, with 61 to 75 years being the most common age group. Falls and traffic accidents were the predominant etiologies. Farmers represented the highest-risk occupational group. Prevention strategies should particularly target fall risk among older adults and electric bicycle–related injuries among young men. These findings highlight the need for tailored injury-prevention measures and underscore the crucial role of rehabilitation in the long-term management of TSCI.

## 1. Introduction

Traumatic spinal cord injury (TSCI) is among the most severe forms of trauma and is typically caused by high-energy mechanisms, resulting in varying degrees of damage to the spinal cord, nerve roots, cauda equina, and other intraspinal structures.^[1]^ Owing to its high disability rate, frequent complications, limited treatment options, and substantial medical and social costs, TSCI represents a major disease burden and an important public health concern worldwide.^[2]^

The global burden of TSCI appears to be increasing in the context of rapid economic development and industrialization. However, effective clinical interventions remain limited and long-term functional recovery is still challenging for many patients.^[3]^ Consequently, primary prevention is likely to be the most effective way to reduce the impact of TSCI. Understanding the epidemiological characteristics of TSCI is essential for identifying high-risk populations, designing targeted prevention strategies, and informing health-care planning.^[4]^ A global survey conducted in 2010 reported that the prevalence of TSCI ranged from 13.1 to 52.2 per million.^[5]^ Annual incidence also varies considerably between regions, with estimates of approximately 40 cases per million in North America,^[6]^ around 15 per million in Western Europe,^[7]^ and 12.1 to 61.6 per million in Asia.^[8]^

In China, reported annual incidences differ markedly by region, including 60.6 per million in Beijing, 23.7 per million in Tianjin, and 61.6 per million in Taiwan.^[8–10]^ Unlike many high-income countries, China currently lacks a national spinal cord injury (SCI) registry.^[11]^ As a result, most available data are derived from single- or multicenter hospital-based studies that primarily describe demographic and injury patterns rather than providing robust incidence estimates.^[12]^ Given the substantial regional variability in TSCI epidemiology, localized studies are necessary to accurately characterize high-risk groups and to develop context-specific preventive measures.

Jinan, the capital of Shandong Province in northern China, is located in the central-eastern region of the country and covers an area of 10,244 km^2^, with a resident population exceeding 9.5 million as of 2024. Jinan hosts multiple leading medical centers and serves as a major referral hub for high-energy trauma from central and western Shandong. Despite its representativeness as an urban-rural integrated city in northern China, no comprehensive epidemiological analysis of TSCI in Jinan has been reported. Specifically, profiling etiology, neurological level and American Spinal Injury Association Impairment Scale (AIS) grade, concomitant injuries, in-hospital complications, treatment patterns, and length of stay can help identify locally dominant injury mechanisms, anticipate acute care and rehabilitation demands, and provide a structured baseline for future studies on prevention and outcomes in this region. Therefore, this study aimed to describe the epidemiological characteristics of TSCI in Jinan over a 10-year period and to provide evidence to support health-care resource planning and the development of targeted injury-prevention strategies.

## 2. Materials and methods

### 2.1. Study design and setting

According to the official website of the Jinan Municipal Health Commission, no population-based TSCI registry system currently exists in Jinan. Therefore, available TSCI data rely primarily on hospital records rather than systematic, multicenter surveillance.

In this retrospective observational study, we reviewed medical records of patients hospitalized with TSCI at the 960th Hospital of Joint Logistics Support Force of PLA and Qilu Hospital of Shandong University between January 2015 and December 2024.

### 2.2. Participants

Patients were eligible if they had a diagnosis of traumatic spinal cord or cauda equina injury sustained within the Jinan area and were subsequently admitted to one of the 2 participating hospitals. TSCI was identified according to the International Classification of Diseases, 10th Revision codes, and neurological impairment was classified using the AIS, grades A to D. Case identification was initially based on International Classification of Diseases, 10th Revision discharge codes suggestive of traumatic spinal cord or cauda equina injury. Eligibility was then confirmed by chart review, including neurological examination documented at admission using the International Standards for Neurological Classification of SCI and AIS grading by trained spine/rehabilitation physicians, and imaging confirmation (computed tomography and/or magnetic resonance imaging) to verify traumatic spinal canal/cord involvement and to define the anatomical level(s) of injury. In routine practice at both hospitals, patients with suspected TSCI undergo emergency assessment, spine computed tomography for bony injury screening, and magnetic resonance imaging when feasible for cord/ligament evaluation, followed by definitive AIS grading after stabilization.

Exclusion criteria were: nontraumatic spinal pathologies (e.g., tumors, myelitis, and intervertebral disc disorders), vertebral fractures without spinal cord involvement, incomplete or unclear clinical records, patients treated only in the emergency department without hospitalization, and prehospital deaths.

### 2.3. Variables and definitions

Collected variables included sex, age, ethnicity, marital status, occupation, education level, time of injury, injury etiology, neurological level of injury, AIS grade at admission, concomitant injuries, in-hospital complications, treatments received, length of hospital stay, and in-hospital mortality (all-cause death during the index hospitalization, with time from admission to death recorded when applicable). Ethnicity was obtained from the standardized demographic information in the medical record.

Patients were classified into 6 age groups: 0 to 15, 16 to 30, 31 to 45, 46 to 60, 61 to 75, and ≥ 76 years. Marital status was categorized as married, unmarried, divorced, or widowed.

Injury etiology included traffic accidents (four-wheeled motor vehicles, electric bicycles, and other vehicles, sex stratification by traffic mechanism was prospectively recorded for electric bicycle-related crashes only; sex-specific mechanism information for other traffic accident categories was not systematically collected in the registry), falls (low-level < 1 meter; high-level ≥ 1 meter),^[13]^ injuries caused by falling objects, machinery-related incidents, and sports-related trauma.

Occupations were categorized as worker, farmer, government employee, student, retiree, military personnel, and others (self-employed and unemployed). Neurological injury levels were grouped anatomically as follows: upper cervical (C1–C2), lower cervical (C3–C7), upper thoracic (T1–T4), mid-thoracic (T5–T9), lower thoracic (T10–T12), upper lumbar (L1–L3), lower lumbar (L4–L5), and sacral (S1–S3). Multilevel injuries were further classified as cervicothoracic (C5–T3), thoracolumbar (T10–L2), and cervicothoracolumbar (C3–L5).

### 2.4. Data collection and management

Data were extracted from the hospital information systems by trained research staff and entered into Microsoft Excel 2019 (Microsoft Corp.). All entries were checked for completeness and internal consistency before analysis.

### 2.5. Statistical analysis

Statistical analyses were performed using Statistical Package for the Social Sciences version 25.0 (IBM Corp.). Descriptive statistics were used to summarize baseline characteristics. Categorical variables are presented as numbers and percentages, and continuous variables as means with standard deviations or medians with interquartile ranges, as appropriate. As an exploratory analysis, associations between categorical variables were evaluated using the chi-square test (or Fisher exact test when appropriate), including etiology versus age group, etiology versus AIS grade, and etiology versus injury level. A 2-sided *P* value < .05 was considered statistically significant.

## 3. Results

A total of 1213 potentially eligible patients were identified from the hospital records of the 960th Hospital of the Joint Logistics Support Force of the PLA and Qilu Hospital of Shandong University between 2015 and 2024. After detailed chart review, 79 patients were excluded, including 48 with nontraumatic spinal cord injuries (36 with disc herniation, 5 with myelitis, and 7 with intraspinal masses), 18 with vertebral fractures without spinal cord involvement, 9 with incomplete clinical records, and 4 with unclear diagnoses. Finally, 1134 patients met the inclusion criteria and were included in the final analysis. The participant selection process is shown in Figure S1.

### 3.1. Demographic characteristics

The demographic characteristics of the 1134 included patients are summarized in Table 1. Of these, 892 (78.7%) were male and 242 (21.3%) were female, yielding a male-to-female ratio of 3.69:1. Ages ranged from 7 months to 93 years (mean [standard deviation] 62.0 [18.6] years; 95% confidence interval, 60.9–63.1). The largest age group was 61 to 75 years (33.3%, n = 377), followed by ≥ 76 years (30.0%, n = 340) and 46 to 60 years (19.8%, n = 224).

**Table 1:** Characteristics of individuals with TSCI from 2015 to 2024.

Years	2015	2016	2017	2018	2019	2020	2021	2022	2023	2024	Total
Age											
0–15	2 (0.18%)	1 (0.09%)	1 (0.09%)	1 (0.09%)	0 (0.0%)	0 (0.0%)	4 (0.35%)	6 (0.53%)	9 (0.79%)	11 (0.97%)	35 (3.09%)
16–30	1 (0.09%)	0 (0.0%)	2 (0.18%)	2 (0.18%)	1 (0.09%)	1 (0.09%)	5 (0.44%)	8 (0.71%)	13 (1.15%)	21 (1.85%)	54 (4.76%)
31–45	3 (0.26%)	1 (0.09%)	3 (0.26%)	3 (0.26%)	2 (0.18%)	2 (0.18%)	11 (0.97%)	14 (1.23%)	31 (2.73%)	34 (3.0%)	104 (9.17%)
46–60	11 (0.97%)	5 (0.44%)	10 (0.88%)	10 (0.88%)	7 (0.62%)	7 (0.62%)	22 (1.94%)	34 (3.0%)	50 (4.41%)	68 (6.0%)	224 (19.75%)
61–75	17 (1.5%)	8 (0.71%)	14 (1.23%)	14 (1.23%)	10 (0.88%)	12 (1.06%)	40 (3.53%)	51 (4.5%)	96 (8.47%)	115 (10.14%)	377 (33.25%)
≥ 76	14 (1.23%)	7 (0.62%)	13 (1.15%)	13 (1.15%)	10 (0.88%)	11 (0.97%)	35 (3.09%)	50 (4.41%)	73 (6.44%)	114 (10.05%)	340 (29.98%)
Gender											
Male	38 (3.35%)	17 (1.5%)	35 (3.09%)	33 (2.91%)	24 (2.12%)	26 (2.29%)	92 (8.11%)	128 (11.29%)	214 (18.87%)	285 (25.13%)	892 (78.66%)
Female	10 (0.88%)	5 (0.44%)	8 (0.71%)	10 (0.88%)	6 (0.53%)	7 (0.62%)	25 (2.2%)	35 (3.09%)	58 (5.11%)	78 (6.88%)	242 (21.34%)
Education level											
Illiterate	1 (0.09%)	0 (0.0%)	0 (0.0%)	0 (0.0%)	0 (0.0%)	0 (0.0%)	0 (0.0%)	0 (0.0%)	0 (0.0%)	0 (0.0%)	1 (0.09%)
Preschool	0 (0.0%)	0 (0.0%)	0 (0.0%)	0 (0.0%)	0 (0.0%)	1 (0.09%)	0 (0.0%)	0 (0.0%)	1 (0.09%)	1 (0.09%)	3 (0.26%)
Elementary school	15 (1.32%)	7 (0.62%)	13 (1.15%)	13 (1.15%)	9 (0.79%)	9 (0.79%)	36 (3.17%)	66 (5.82%)	86 (7.58%)	102 (8.99%)	356 (31.39%)
Middle school	18 (1.59%)	8 (0.71%)	16 (1.41%)	17 (1.5%)	11 (0.97%)	12 (1.06%)	43 (3.79%)	49 (4.32%)	103 (9.08%)	139 (12.26%)	416 (36.68%)
High school	11 (0.97%)	5 (0.44%)	11 (0.97%)	9 (0.79%)	8 (0.71%)	8 (0.71%)	29 (2.56%)	36 (3.17%)	65 (5.73%)	96 (8.47%)	278 (24.51%)
College or more	3 (0.26%)	2 (0.18%)	3 (0.26%)	4 (0.35%)	2 (0.18%)	3 (0.26%)	9 (0.79%)	12 (1.06%)	17 (1.5%)	25 (2.2%)	80 (7.05%)
Occupation											
Farmer	21 (1.85%)	11 (0.97%)	20 (1.76%)	17 (1.5%)	12 (1.06%)	14 (1.23%)	46 (4.06%)	67 (5.91%)	116 (10.23%)	163 (14.37%)	487 (42.95%)
Worker	7 (0.62%)	3 (0.26%)	6 (0.53%)	7 (0.62%)	4 (0.35%)	6 (0.53%)	17 (1.5%)	26 (2.29%)	42 (3.7%)	50 (4.41%)	168 (14.81%)
Retired	2 (0.18%)	0 (0.0%)	2 (0.18%)	3 (0.26%)	1 (0.09%)	2 (0.18%)	5 (0.44%)	6 (0.53%)	10 (0.88%)	15 (1.32%)	46 (4.06%)
Government	1 (0.09%)	1 (0.09%)	1 (0.09%)	1 (0.09%)	1 (0.09%)	1 (0.09%)	3 (0.26%)	4 (0.35%)	6 (0.53%)	9 (0.79%)	28 (2.47%)
Student	1 (0.09%)	0 (0.0%)	1 (0.09%)	1 (0.09%)	1 (0.09%)	1 (0.09%)	2 (0.18%)	4 (0.35%)	6 (0.53%)	10 (0.88%)	27 (2.38%)
Soldier	0 (0.0%)	0 (0.0%)	0 (0.0%)	0 (0.0%)	0 (0.0%)	0 (0.0%)	1 (0.09%)	1 (0.09%)	2 (0.18%)	3 (0.26%)	7 (0.62%)
Others	16 (1.41%)	7 (0.62%)	13 (1.15%)	14 (1.23%)	11 (0.97%)	9 (0.79%)	43 (3.79%)	55 (4.85%)	90 (7.94%)	113 (9.96%)	371 (32.72%)
Etiology											
Traffic accidents	19 (1.68%)	9 (0.79%)	17 (1.50%)	19 (1.68%)	12 (1.06%)	14 (1.23%)	45 (3.97%)	63 (5.56%)	104 (9.17%)	159 (14.02%)	461 (40.65%)
Low fall	12 (1.06%)	7 (0.62%)	12 (1.06%)	9 (0.79%)	8 (0.71%)	8 (0.71%)	28 (2.47%)	47 (4.14%)	80 (7.05%)	82 (7.23%)	293 (25.84%)
High fall	10 (0.88%)	4 (0.35%)	8 (0.71%)	9 (0.79%)	5 (0.44%)	6 (0.53%)	29 (2.56%)	31 (2.73%)	51 (4.5%)	76 (6.7%)	229 (20.19%)
Sport	3 (0.26%)	1 (0.09%)	2 (0.18%)	3 (0.26%)	2 (0.18%)	3 (0.26%)	7 (0.62%)	11 (0.97%)	15 (1.32%)	20 (1.76%)	67 (5.91%)
Falling objects	3 (0.26%)	1 (0.09%)	2 (0.18%)	2 (0.18%)	1 (0.09%)	1 (0.09%)	5 (0.44%)	8 (0.71%)	16 (1.41%)	19 (1.68%)	58 (5.11%)
Machinery-injury	1 (0.09%)	0 (0.0%)	2 (0.18%)	1 (0.09%)	2 (0.18%)	1 (0.09%)	3 (0.26%)	3 (0.26%)	6 (0.53%)	7 (0.62%)	26 (2.29%)
Marital status											
Married	45 (3.97%)	21 (1.85%)	40 (3.53%)	39 (3.44%)	27 (2.38%)	30 (2.65%)	109 (9.61%)	153 (13.49%)	253 (22.31%)	340 (29.98%)	1057 (93.21%)
Unmarried	2 (0.18%)	1 (0.09%)	2 (0.18%)	3 (0.26%)	2 (0.18%)	3 (0.26%)	6 (0.53%)	7 (0.62%)	16 (1.41%)	18 (1.59%)	60 (5.29%)
Widowed	1 (0.09%)	0 (0.0%)	1 (0.09%)	1 (0.09%)	1 (0.09%)	0 (0.0%)	1 (0.09%)	3 (0.26%)	3 (0.26%)	5 (0.44%)	16 (1.41%)
Divorced	0 (0.0%)	0 (0.0%)	0 (0.0%)	0 (0.0%)	0 (0.0%)	0 (0.0%)	1 (0.09%)	0 (0.0%)	0 (0.0%)	0 (0.0%)	1 (0.09%)
Ethnic groups											
Han Chinese	48 (4.23%)	22 (1.94%)	43 (3.79%)	43 (3.79%)	30 (2.65%)	32 (2.82%)	116 (10.23%)	162 (14.29%)	270 (23.81%)	361 (31.83%)	1127 (99.38%)
Hui ethnic group	0 (0.0%)	0 (0.0%)	0 (0.0%)	0 (0.0%)	0 (0.0%)	0 (0.0%)	1 (0.09%)	0 (0.0%)	1 (0.09%)	1 (0.09%)	3 (0.26%)
Manchu	0 (0.0%)	0 (0.0%)	0 (0.0%)	0 (0.0%)	0 (0.0%)	1 (0.09%)	0 (0.0%)	0 (0.0%)	0 (0.0%)	1 (0.09%)	2 (0.18%)
Mongol	0 (0.0%)	0 (0.0%)	0 (0.0%)	0 (0.0%)	0 (0.0%)	0 (0.0%)	0 (0.0%)	1 (0.09%)	1 (0.09%)	0 (0.0%)	2 (0.18%)

Others included unemployed individuals and self-employed individuals.

TSCI = traumatic spinal cord injury.

Most patients had relatively low educational attainment: 416 (36.7%) had completed junior high school, and 360 (31.7%) had primary education or less, including 1 (0.1%) illiterate individual and 3 (0.3%) preschool children. Only 80 (7.1%) patients had a college degree or higher.

Farmers constituted the largest occupational group (42.9%, n = 487), followed by the “others” category (self-employed and unemployed; 32.7%, n = 371) and workers (14.8%, n = 168). Government employees, students, and military personnel accounted for relatively small proportions (Table 1).

Most patients were married (93.2%, n = 1057). The majority were of Han Chinese (99.4%, n = 1127), with small proportions of Hui ethnic group (0.3%, n = 3), Manchu (0.2%, n = 2), and Mongol (0.2%, n = 2) ethnicity.

### 3.2. Etiology of injury

The causes of injury are detailed in Table 1. Falls were the leading cause of TSCI (46.0%, n = 522), including low-level falls (< 1 meter; 25.8%, n = 293) and high-level falls (≥ 1 meter; 20.2%, n = 229), followed by traffic accidents (40.7%, n = 461). Less frequent causes included sports-related injuries (5.9%, n = 67), injuries from falling objects (5.1%, n = 58), and machinery-related injuries (2.3%, n = 26).

**Figure 1.**
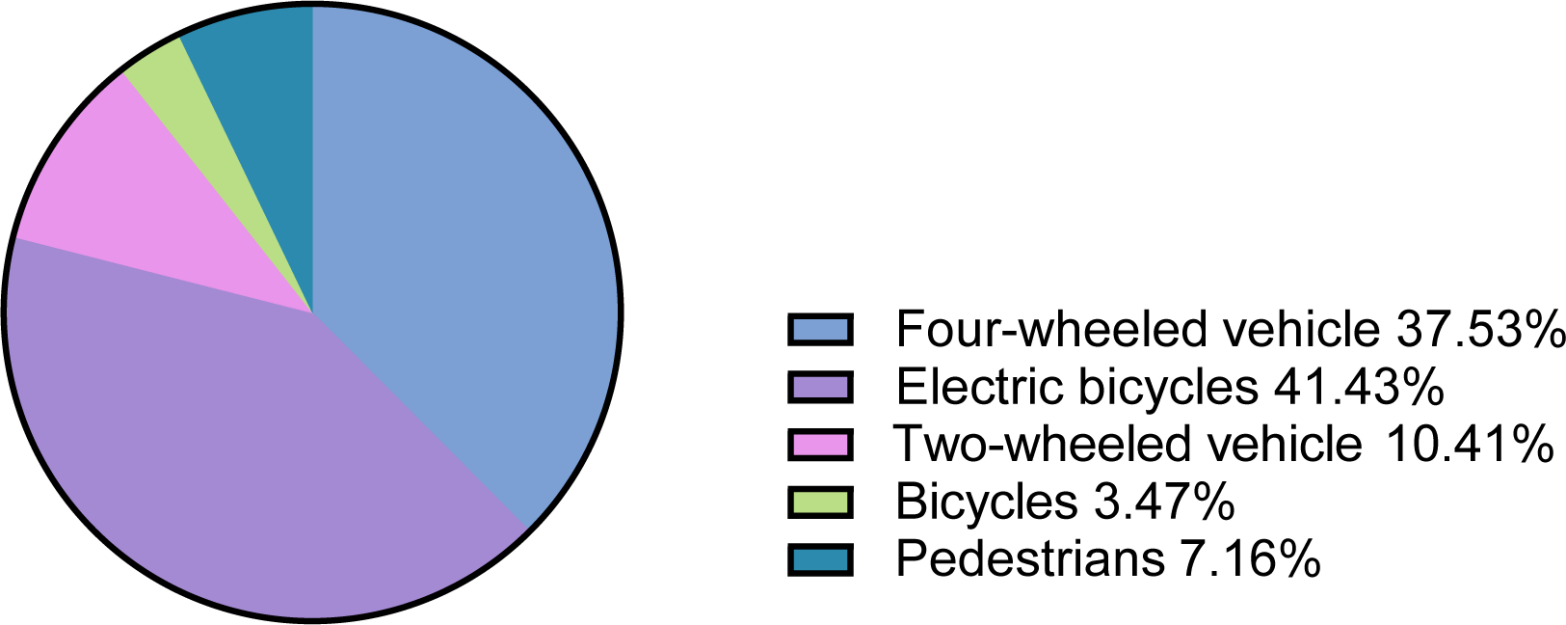
Details of traffic accidents.

Among traffic-related injuries, electric bicycles were the most common mechanism (41.4%, n = 191), followed by 4-wheeled motor vehicles (37.5%, n = 173), with fewer cases involving 2-wheeled motor vehicles, bicycles, and pedestrians (Fig. 1). Among female patients, traffic accidents were the leading etiology (113/242, 46.7%) (Table 2). Among traffic accidents (n = 461), electric bicycles accounted for 41.43% (191/461) (Fig. 1). The male-to-female ratio for electric bicycle–related injuries was 3.34:1 (Table S1).

**Table 2:** Analysis of the etiologies and gender among individuals with TSCI from 2015 to 2024.

Etiologies	Gender	
	Male	Female
Traffic accident	348 (39.01%)	113 (46.69%)
Low fall	228 (25.56%)	65 (26.86%)
High fall	203 (22.76%)	26 (10.74%)
Falling objects	53 (5.94%)	5 (2.07%)
Machinery-injury	19 (2.13%)	7 (2.89%)
Sport	41 (4.60%)	26 (10.74%)

Percentages are calculated within each sex (male, n = 892; female, n = 242).

TSCI = traumatic spinal cord injury.

Table 3 shows the distribution of injury etiologies by age group. The 61 to 75-year group accounted for the largest proportion of TSCI cases (33.3%, n = 377), followed by the ≥ 76-year group (30.0%, n = 340). Across age strata, traffic accidents and falls remained the 2 predominant etiologies, with low-level falls contributing a larger share in the 2 oldest groups. The etiology distribution differed significantly across age groups (*χ*^2^ = 93.73, df = 25, *P* < .001).

**Table 3:** Analysis of the etiologies and age distribution among individuals with TSCI from 2015 to 2024.

Etiologies	Age					
	0–15	16–30	31–45	46–60	61–75	≥ 76
Traffic accident	8 (0.71%)	22 (1.94%)	39 (3.44%)	94 (8.29%)	166 (14.64%)	132 (11.64%)
Low fall	5 (0.44%)	6 (0.53%)	18 (1.59%)	43 (3.79%)	95 (8.38%)	126 (11.11%)
High fall	11 (0.97%)	17 (1.50%)	25 (2.20%)	60 (5.29%)	67 (5.91%)	49 (4.32%)
Falling objects	1 (0.09%)	3 (0.26%)	11 (0.97%)	12 (1.06%)	23 (2.03%)	8 (0.71%)
Machinery-injury	2 (0.18%)	2 (0.18%)	3 (0.26%)	6 (0.53%)	11 (0.97%)	2 (0.18%)
Sport	8 (0.71%)	4 (0.35%)	8 (0.71%)	9 (0.79%)	15 (1.32%)	23 (2.03%)

TSCI = traumatic spinal cord injury.

### 3.3. Neurological level and severity of injury

**Figure 2.**
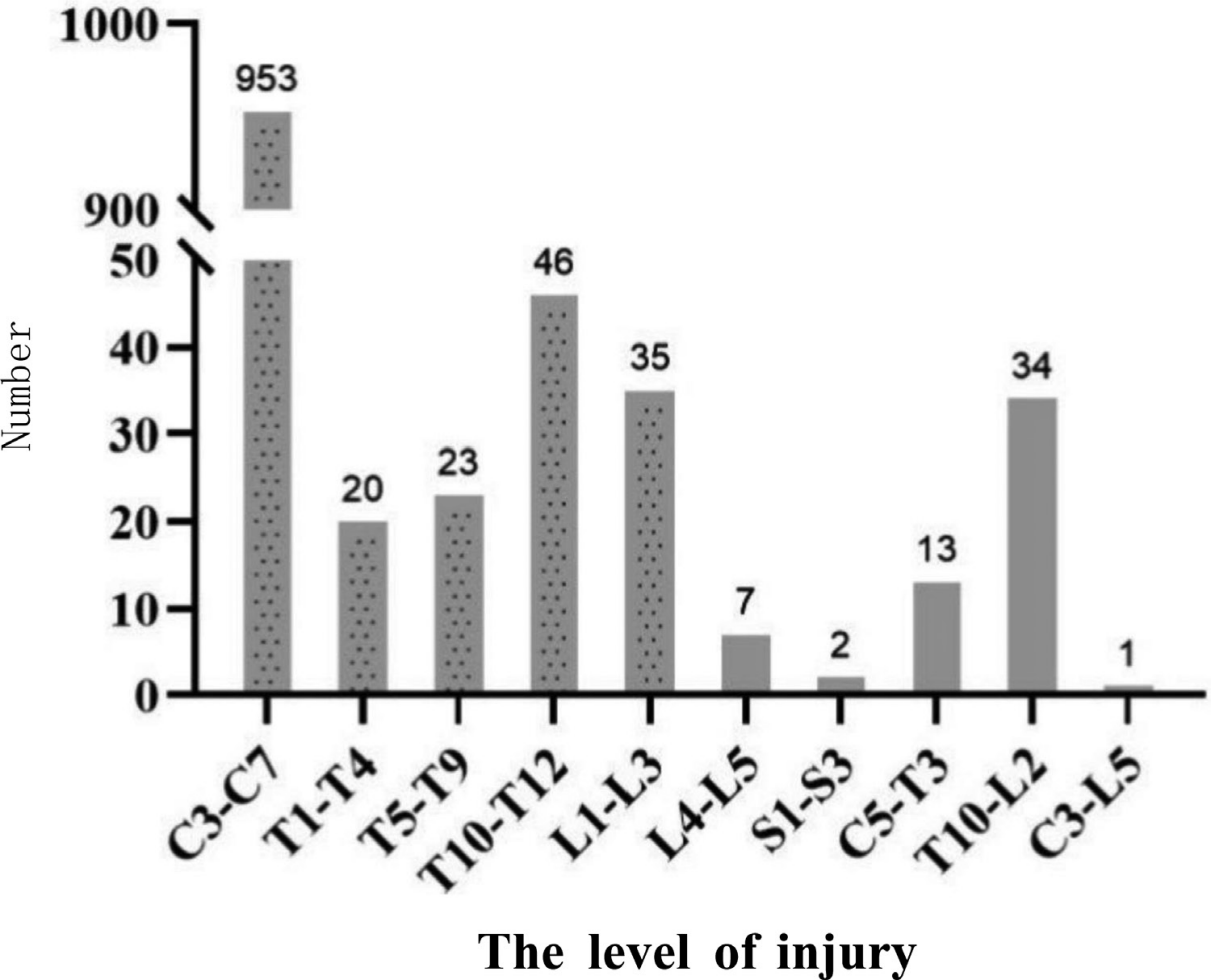
Distribution histogram of the injury level of the patients.

Neurological levels of injury showed a unimodal distribution, with a clear peak at the cervical spine (Fig. 2). According to the AIS, 387 patients (34.1%) were classified as Grade A, 250 (22.0%) as Grade B, 244 (21.5%) as Grade C, and 253 (22.3%) as Grade D.

The relationship between neurological level and AIS grade is shown in Table 4. Most cervical injuries were classified as Grade A (26.0%, n = 295) or Grade D (20.2%, n = 229). Among thoracic injuries, Grade A also predominated (5.6%, n = 63). AIS grade distribution differed across injury levels (χ^2^ = 43.85, df = 9, *P* < .001) (Table 4).

**Table 4:** Comparison of the degrees and segments of the injury among individuals with TSCI.

ASIA scale	The level of injury			
	Cervical cord (%)	Thoracic cord (%)	Lumbar cord (%)	Sacral cord (%)
A	295 (26.01%)	63 (5.56%)	29 (2.56%)	0 (0.00%)
B	209 (18.43%)	32 (2.82%)	8 (0.71%)	1 (0.09%)
C	220 (19.40%)	13 (1.15%)	11 (0.97%)	0 (0.00%)
D	229 (20.19%)	10 (0.88%)	13 (1.15%)	1 (0.09%)

ASIA = American Spinal Injury Association, TSCI = traumatic spinal cord injury.

Associations between etiology and neurological severity are presented in Table 5. Traffic accidents and high-level falls contributed a larger proportion of AIS A injuries, whereas low-level falls were more frequently associated with incomplete injuries, particularly AIS C and AIS D patterns. Etiology was significantly associated with AIS grade (*χ*^2^ = 128.88, df = 15, *P* < .001). Injury level also varied by etiology, with cervical injuries mainly caused by traffic accidents and low-level falls, whereas thoracic injuries were more commonly related to traffic accidents and high-level falls (*χ*^2^ = 101.34, df = 15, *P* < .001).

**Table 5:** Analysis of the causes, segments, and degrees of injury among individuals with TSCI.

Etiologies	ASIA scale			
	A (%)	B (%)	C (%)	D (%)
Traffic accident	173 (15.26%)	99 (8.73%)	93 (8.20%)	96 (8.47%)
Low fall	43 (3.79%)	67 (5.91%)	95 (8.38%)	88 (7.76%)
High fall	123 (10.85%)	55 (4.85%)	29 (2.56%)	22 (1.94%)
Falling objects	23 (2.03%)	10 (0.88%)	7 (0.62%)	18 (1.59%)
Machinery-injury	12 (1.06%)	4 (0.35%)	6 (0.53%)	4 (0.35%)
Sport	13 (1.15%)	15 (1.32%)	14 (1.23%)	25 (2.20%)
Etiologies	The level of injury			
	Cervical cord (%)	Thoracic cord (%)	Lumbar cord (%)	Sacral cord (%)
Traffic accident	418 (36.86%)	32 (2.82%)	11 (0.97%)	0 (0.00%)
Low fall	268 (23.63%)	17 (1.50%)	7 (0.62%)	1 (0.09%)
High fall	154 (13.58%)	47 (4.14%)	28 (2.47%)	0 (0.00%)
Falling objects	41 (3.62%)	11 (0.97%)	6 (0.53%)	0 (0.00%)
Machinery-injury	21 (1.85%)	4 (0.35%)	1 (0.09%)	0 (0.00%)
Sport	51 (4.50%)	7 (0.62%)	8 (0.71%)	1 (0.09%)

ASIA = American Spinal Injury Association, TSCI = traumatic spinal cord injury.

### 3.4. Time to hospitalization and treatment

The time from injury to hospital admission ranged from 0.5 to 1004 hours, with a median of 13.5 hours. Some patients were transported directly by ambulance, whereas others initially presented to lower-level medical institutions before being referred.

Length of hospitalization ranged from 1 to 472 days, with a median of 21 days (interquartile range 8–34 days). Surgical treatment was performed in 88.9% of patients (1009/1134), including procedures such as laminoplasty, spinal decompression, fusion, and internal fixation. Additionally, 746 patients (65.8%) received rehabilitation therapy, 664 (58.6%) received traditional therapies (traditional Chinese medicine–based adjuvant care, e.g., acupuncture/electroacupuncture, moxibustion, cupping, and tuina/manual therapy), and 242 (21.3%) underwent hyperbaric oxygen therapy (Table 6). Fourteen in-hospital deaths occurred, and the mean time from admission to death was 24 days.

**Table 6:** The treatment options for persons with TSCI.

Treatment options	Numbers (%)
Surgery	
Yes	1009 (88.98%)
No	125 (11.02%)
Rehabilitation therapy	
Yes	746 (65.78%)
No	388 (34.22%)
Traditional therapy	
Yes	664 (58.55%)
No	470 (41.44%)
Hyperbaric oxygen therapy	
Yes	242 (21.34%)
No	892 (78.66%)
Assistive devices	
Yes	445 (39.24%)
No	689 (60.76%)
Medicine	
Yes	153 (13.49%)
No	981 (86.51%)

TSCI = traumatic spinal cord injury.

### 3.5. Associated injuries and clinical complications

Common associated injuries included pulmonary contusions and hemopneumothorax (28.9%, n = 328), rib or limb fractures (25.3%, n = 287), isolated limb fractures (14.4%, n = 163), craniocerebral injuries (13.6%, n = 154), and pelvic fractures (11.5%, n = 130). No associated injuries were reported in 177 patients (15.6%).

Clinical complications occurred in 500 patients (44.1%). The most common complications were pulmonary infections (16.5%, n = 187), urinary tract infections (13.6%, n = 154), hyponatremia (6.8%, n = 77), pressure ulcers (3.2%, n = 36), deep vein thrombosis (2.4%, n = 27), and other complications (1.7%, n = 19).

## 4. Discussion

A recent systematic review of 21 studies from China showed significant regional variations in SCI epidemiology, predominantly concentrated in economically developed provinces in eastern and southern China.^[14]^ This underscores the necessity of customized prevention strategies tailored to specific regional demographics and socioeconomic contexts.

This study provides the first comprehensive epidemiological analysis of TSCI in Jinan, China, based on retrospective data from 2 tertiary hospitals spanning nearly a decade. Although retrospective studies inherently subject to the risk of data loss, reviewing extensive hospital records minimized missing information.

Our findings revealed a male-to-female ratio of approximately 3.69:1, consistent with ratios of 2.4:1 to 4.8:1 reported in other regional studies.^[12,15,16]^ This gender disparity likely results from men typically engaging in higher-risk occupations, while women often undertake lower-risk household tasks.

The mean age in this cohort (62.0 years) was higher than that reported in several other Chinese regions.^[17–19]^ This pattern is consistent with the age distribution in our dataset, where the 61 to 75-year group was the largest and patients aged ≥ 76 years also comprised a substantial proportion. Jinan and its referral catchment include large urban-rural integrated areas with an aging population; older adults are more prone to low-energy mechanisms such as low-level falls and may also have preexisting degenerative changes that increase vulnerability to SCI. These factors likely contribute to the observed older age profile and underscore the need to prioritize fall-prevention strategies in older adults.

Additionally, China’s rapidly aging population is associated with an increased incidence of SCI among older adults. Elderly individuals often have comorbid conditions, such as osteoporotic fractures and degenerative spinal diseases such as cervical stenosis or ossification of the posterior longitudinal ligament, increasing their vulnerability to spinal injuries even following minor trauma.^[20,21]^

In our study, farmers represented the largest occupational group affected (42.9%), aligning with studies from Chongqing, Tianjin, and Heilongjiang.^[13,22,23]^ This trend reflects low educational attainment and high exposure to hazardous labor among rural agricultural workers in Shandong, highlighting an urgent need for targeted occupational safety interventions.

Most participants were married, which is consistent with the predominance of TSCI among middle-aged individuals. In China, marriage typically occurs during this age range, explaining the observed demographic patterns.

Falls (46.0%) and traffic accidents (40.7%) were identified as the primary injury etiologies, consistent with earlier reports.^[9,12,13]^ While traffic accidents dominate TSCI causes in developed nations, high-level falls (often linked to construction work) prevail in developing countries.^[1,2]^ This likely reflects the pace of China’s industrialization and extensive infrastructure development. Recent improvements in road conditions, public education, and stricter enforcement against drunk driving might explain why motor vehicle accidents did not surpass falls as the leading cause of TSCI in Jinan. Gunshot injuries were not observed, consistent with China’s strict firearm regulations.

High-energy trauma (traffic accidents and high-level falls) predominated among younger individuals, whereas low-energy injuries (low-level falls) were more common in older adults.^[21]^ Etiological patterns of TSCI differ by country and region, with traffic accidents most prevalent in high-income nations (41.6%) and lower proportions reported in middle-income (40.7%) and low-income countries (27.2%).^[24]^ Regions with aging populations, (such as Western Europe and high-income Asia-Pacific areas) report falls as the leading cause.^[7]^

Interestingly, electric bicycles emerged as the most frequent traffic-related injury mechanism (41.4%), surpassing cars (37.5%). Electric bicycles have become a dominant mode of transportation in Jinan, a long-established city where metro coverage is still expanding and road congestion is common. Their convenience and low barrier to use may increase exposure across age groups, including both young male riders and older adults in an aging population. Notably, electric bicycle–related injuries may involve different roles (rider versus pedestrian/other road-user struck) and may vary by age. Future studies should prospectively capture road-user role, helmet use, and crash context to inform age-specific prevention policies.

Cervical injuries accounted for 84.0% of cases, which aligns with prior studies that reported cervical spine involvement in 55 to 75% of SCI.^[23,25]^ Our detailed anatomical analysis indicated all cervical injuries occurred between C3 and C7, distinctly contrasting the bimodal distribution (cervical and thoracolumbar peaks) found elsewhere.^[13,26]^ The absence of upper cervical (C1–C2) injuries may reflect their high fatality rate, reducing hospital-based representation. Multilevel injuries were uncommon (cervicothoracic, 1.2%; thoracolumbar, 3.0%; cervicothoracolumbar, 0.1%), and no lumbosacral multilevel injuries occurred.

The distribution of American Spinal Injury Association impairment grades was as follows: Grade A (34.1%), Grade B (22.0%), Grade C (21.5%), and Grade D (22.3%). High-energy traumas (traffic accidents and high-level falls) frequently resulted in complete (Grade A) injuries, whereas low-energy falls primarily produced incomplete (Grade C) injuries. Individuals with Grade A injuries are particularly vulnerable to depression and suicide,^[27]^ emphasizing the importance of psychosocial interventions by caregivers and healthcare professionals.

We found that neurological level and AIS grade were significantly associated (*χ*^2^ = 43.85, df = 9, *P* < .001), with cervical injuries accounting for the majority of complete injuries in this cohort. Given the high incidence of complete injuries, enhancing prehospital emergency responses is crucial for timely intervention.

Clinical complications affected 44.1% of participants, most commonly pulmonary infections (16.5%), urinary tract infections (13.6%), hyponatremia (6.8%), and pressure ulcers (3.2%), comparable to reports from northwest China^[20]^ and Italy.^[28]^ Pulmonary and urinary tract infections often reflect suboptimal hospital care and are exacerbated by prolonged immobility, preexisting respiratory diseases, and impaired respiratory muscle function, especially with high cervical injuries (C5 and above), where infection rates reach 90%.^[29,30]^ This emphasizes comprehensive clinical care to reduce complications and the length of hospitalization.

This study has several limitations. First, it is hospital-based and may not fully represent the entire population. The absence of a regional SCI registry in Shandong limits generalizability. Second, retrospective analyzes risk incomplete data. Third, sex-stratified traffic accident mechanism data were not systematically collected beyond electric bicycle crashes, precluding comparisons across all traffic mechanisms. Fourth, complications and treatments might be underreported. Finally, prehospital deaths were excluded, which potentially underestimates injury prevalence.

## 5. Conclusion

This 10-year hospital-based epidemiological study from Jinan, northern China, identified a distinct TSCI pattern characterized by a predominance of male patients and injuries mainly caused by falls and electric bicycle–related traffic accidents. Middle-aged and older men engaged in high-risk outdoor or manual labor, as well as younger men working in food delivery and courier services, emerged as key high-risk groups who should be prioritized in future prevention efforts.

At the population level, injury prevention may be strengthened by improving urban infrastructure (such as optimizing road networks and installing effective physical separation between motor vehicles, electric bicycles, and pedestrians), implementing tailored safety education programs for different demographic and occupational groups, and enforcing legislation mandating the use of personal protective equipment and helmets in high-risk occupations, together with stricter enforcement of road traffic regulations.

Comprehensive management of TSCI (including timely acute care, prevention and treatment of complications, and long-term rehabilitation) should be further emphasized to improve functional outcomes and quality of life. The present findings provide foundational evidence to support multifaceted, region-specific prevention strategies in Jinan and similar urban-rural integrated settings, and they highlight the need for future research to evaluate targeted interventions and to optimize models of TSCI care.

## Acknowledgments

We sincerely thank Professor Yanan Liu, an epidemiologist, for providing the statistical support.

## Author contributions

**Data curation:** Linyang Song.

**Formal analysis:** Linyang Song.

**Investigation:** Xin Xu.

**Methodology:** Xin Xu.

**Resources:** Ang Li, Ruoxian Song.

**Software:** Ang Li, Junlin Han, Ruoxian Song.

**Supervision:** Ang Li, Junlin Han, Ruoxian Song.

**Validation:** Ruoxian Song.

**Visualization:** Ruoxian Song.

**Writing – original draft:** Kun Wang.

**Writing – review & editing:** Kun Wang.




