# Supplementary material for: Traumatic spinal cord injury in Jinan, China: A 10-year hospital-based retrospective observational study of 1134 cases
Source: Medicine (Baltimore). 2026 May 29;105(22):e49039. doi: 10.1097/MD.0000000000049039 (PMC13225528; doi:10.1097/MD.0000000000049039)
Supplement: Supplementary file 1 [file medi-105-e49039-s001.pptx]

## Slide 1
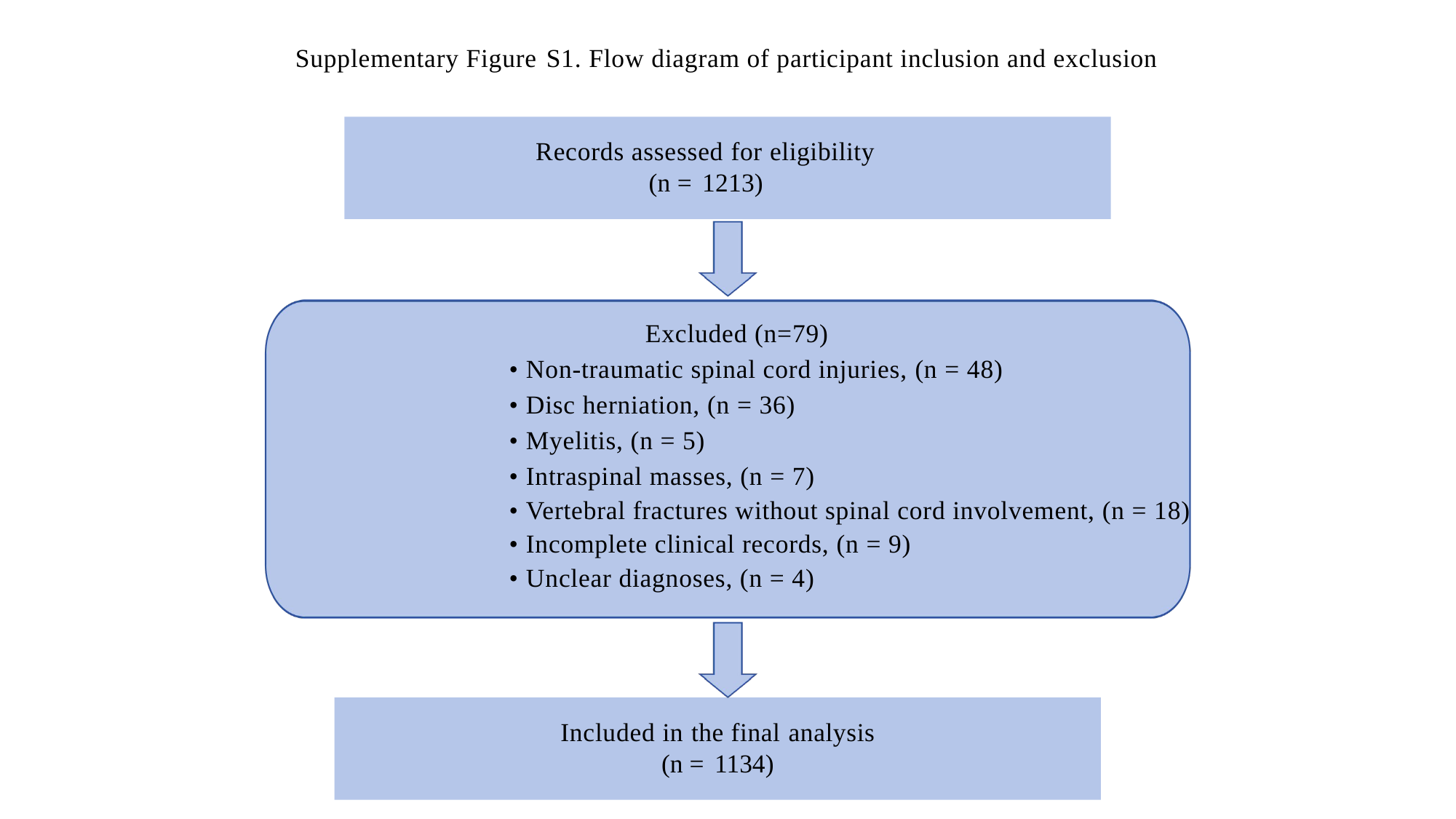

Supplementary Figure S1. Flow diagram of participant inclusion and exclusion
Records assessed for eligibility
(n = 1213)
Excluded (n=79)
• Non-traumatic spinal cord injuries, (n = 48)
• Disc herniation, (n = 36)
• Myelitis, (n = 5)
• Intraspinal masses, (n = 7)
• Vertebral fractures without spinal cord involvement, (n = 18)
• Incomplete clinical records, (n = 9)
• Unclear diagnoses, (n = 4)
Included in the final analysis
(n = 1134)
