# Supplementary material for: Traumatic spinal cord injury in Jinan, China: A 10-year hospital-based retrospective observational study of 1134 cases
Source: Medicine (Baltimore). 2026 May 29;105(22):e49039. doi: 10.1097/MD.0000000000049039 (PMC13225528; doi:10.1097/MD.0000000000049039)
Supplement: Supplementary file 2 [file medi-105-e49039-s002.docx]

**Supplementary Table S1. Traffic accident mechanisms and sex distribution**

| **Mechanism** | **Total, n (%)** | **Male, n** | **Female, n** |
| --- | --- | --- | --- |
| Four-wheeled vehicle | 173 (37.53%) | Not collected | Not collected |
| Electric bicycles | 191 (41.43%) | 147 | 44 |
| Two-wheeled vehicle | 48 (10.41%) | Not collected | Not collected |
| Bicycles | 16 (3.47%) | Not collected | Not collected |
| Pedestrians | 33 (7.16%) | Not collected | Not collected |

Note: Percentages for mechanisms are calculated among traffic accidents (n=461). Sex-specific mechanism data were prospectively recorded for electric bicycle–related crashes only; sex stratification for other traffic mechanisms was not systematically collected in the registry.
